# Synthesis of poly(1,2-glycerol carbonate)–paclitaxel conjugates and their utility as a single high-dose replacement for multi-dose treatment regimens in peritoneal cancer†
†Electronic supplementary information (ESI) available. See DOI: 10.1039/c7sc03501b


**DOI:** 10.1039/c7sc03501b

**Published:** 2017-10-20

**Authors:** Iriny Ekladious, Rong Liu, Heng Zhang, Daniel H. Foil, Daniel A. Todd, Tyler N. Graf, Robert F. Padera, Nicholas H. Oberlies, Yolonda L. Colson, Mark W. Grinstaff

**Affiliations:** a Departments of Biomedical Engineering and Chemistry , Boston University , Boston , MA 02215 , USA . Email: mgrin@bu.edu; b Department of Surgery , Brigham and Women's Hospital , Boston , MA 02215 , USA . Email: ycolson@bwh.harvard.edu; c Department of Chemistry and Biochemistry , University of North Carolina at Greensboro , Greensboro , NC 27402 , USA; d Department of Pathology , Brigham and Women's Hospital , Boston , MA 02215 , USA

## Abstract

Current chemotherapeutic dosing strategies are limited by the toxicity of anticancer agents and therefore rely on multiple low-dose administrations. As an alternative, we describe a novel sustained-release, biodegradable polymeric nanocarrier as a single administration replacement of multi-dose paclitaxel (PTX) treatment regimens. The first synthesis of poly(1,2-glycerol carbonate)-*graft*-succinic acid-paclitaxel (PGC–PTX) is described, and its use enables high, controlled PTX loadings of up to 74 wt%. Moreover, the polymer backbone is composed of biocompatible building blocks—glycerol and carbon dioxide. When formulated as nanoparticles (NPs), PGC–PTX NPs exhibit PTX concentrations >15 mg mL^–1^, sub-100 nm diameters, narrow dispersity, storage stability for up to 6 months, and sustained and controlled PTX release kinetics over an extended period of 70 days. A safely administered single dose of PGC–PTX NPs contains more PTX than the median lethal dose of standard PTX. In murine models of peritoneal carcinomatosis, in which the clinical implementation of multi-dose intraperitoneal (IP) treatment regimens is limited by catheter-related complications, PGC–PTX NPs exhibit improved safety at high doses, tumor localization, and efficacy even after a single IP injection, with comparable curative effect to PTX administered as a multi-dose IP treatment regimen.

## Abstract 2

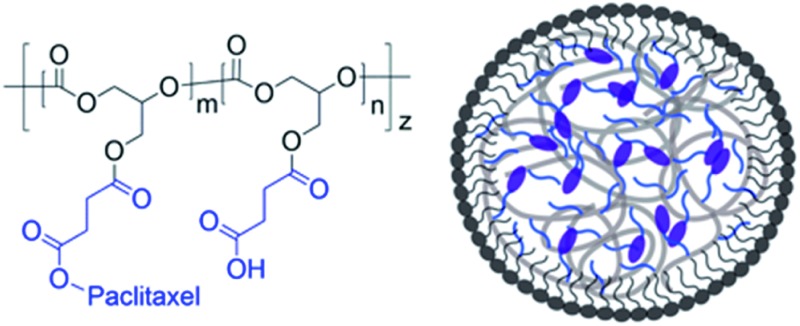
A high drug-density, biodegradable polymeric nanocarrier replaces multi-dose paclitaxel treatment regimens.

## Introduction

Cancer continues to be one of the leading causes of mortality worldwide.^1^,^2^ While cytotoxic chemotherapeutics are a mainstay in the treatment of cancer, the optimization of dosing schedules remains a challenge due to the relatively low therapeutic index of many antineoplastic agents.^3^,^4^ In fact, the dosing of an anticancer agent is typically determined in a phase 1 study, in which the end-point is dose-limiting toxicity rather than efficacy. Although continuous multi-day infusion improves the therapeutic index of several chemotherapeutics, its implementation remains challenging, especially in the local treatment of peritoneal cancers.^4^–^6^ Additionally, control of both drug level and duration of treatment is difficult to achieve *via* current intermittent bolus dosing. Thus, alternative delivery strategies, especially those that are safe and facile to implement (*i.e.*, single administration), are of significant interest.

Nanoparticle (NP) drug delivery systems possess several advantages over conventional small molecule chemotherapeutics.^7^–^15^ Such nanocarriers enhance the delivery of hydrophobic agents, and afford controlled and sustained drug release, thereby increasing the efficacy of many anticancer agents in preclinical animal models. Importantly, NP drug delivery systems provide an opportunity to enhance chemotherapeutic dosing by enabling the safe delivery of higher doses of anti-cancer agents—a topic underexplored in the area of nanomedicine. Of note, one of the advantages of albumin-bound (nab-) paclitaxel (PTX) ABI-007 (Abraxane) is the ability to safely administer doses 70% greater than the PTX standard of care.^16^ Polymeric NPs, in particular, demonstrate significant advantages as a result of their solid nature.^11^,^17^ However, polymeric carriers with physically entrapped agents suffer from low drug loading and significant burst release.^18^ Alternatively, polymer–drug conjugate NPs minimize or eliminate the problem of burst release, while additionally providing the ability to incorporate drugs at high, predefined loadings with specifically engineered release kinetics.^11^,^19^,^20^

PTX is one of the most widely used chemotherapeutic agents for a variety of solid malignancies, including lung, pancreatic, ovarian, breast, and peritoneal mesothelioma. Despite its widespread use, PTX dosing is sub-optimal due to its low therapeutic index, poor solubility, rapid systemic clearance, and limited tumor exposure.^21^–^23^ As a result of its poor aqueous solubility (0.3 μg mL^–1^), PTX is commonly delivered in a cremophor EL/ethanol (1 : 1 v/v; C/E) excipient, which is known to cause adverse side-effects and severe hypersensitivity reactions.^24^,^25^ Thus, alternative delivery methods are of keen interest. Currently under clinical investigation, a water-soluble macromolecular poly(l-glutamic acid)–PTX conjugate (PTX poliglumex) increases PTX solubilization (9 mg mL^–1^) while exhibiting an improved safety profile and enhanced tumor exposure compared to PTX-C/E.^26^ Previously, polymer–PTX conjugate NP delivery systems have demonstrated high loading capacities (5–65 wt%), improved PTX aqueous concentrations, and controlled and sustained PTX release kinetics for up to 10 days.^19^,^20^,^27^–^32^ However, systems that deliver PTX over prolonged periods of time are lacking.

Motivated by these findings, we herein report the first example of a nanocarrier in which PTX is incorporated at high, controlled loadings of up to 74 wt%, and in which PTX concentrations are improved by >50 000 fold (>15 mg mL^–1^) compared to PTX solubility in aqueous solution. Reducing carrier material and maximizing drug content (*i.e.* optimizing drug/material efficiency) departs from the majority of PTX drug carriers which typically achieve <10 wt% drug loading, and therefore require the administration of large amounts of carrier material to achieve a given dose. Moreover, we use poly(1,2-glycerol carbonate) (PGC) as the novel polymer scaffold for PTX attachment as the PGC backbone is readily degradable and biocompatible, consisting of only glycerol and carbon dioxide.^33^,^34^ For this reason, polymers based on glycerol are of significant interest for drug delivery, tissue engineering, and tissue coating applications.^35^–^38^ The polymer's functionalizable pendant primary hydroxy provides a site for PTX conjugation *via* a succinic acid linker in order to give poly(1,2-glycerol carbonate)-*graft*-succinic acid-paclitaxel (PGC–PTX). We demonstrate that PGC–PTX NPs possess sub-100 nm diameters, narrow dispersity, high storage stability, sustained and controlled release kinetics, tunable *in vitro* potency, improved *in vivo* safety at high doses, *in vivo* intraperitoneal (IP) tumor localization, and *in vivo* efficacy even after a single IP injection.

## Results and discussion

**Fig. 1:**
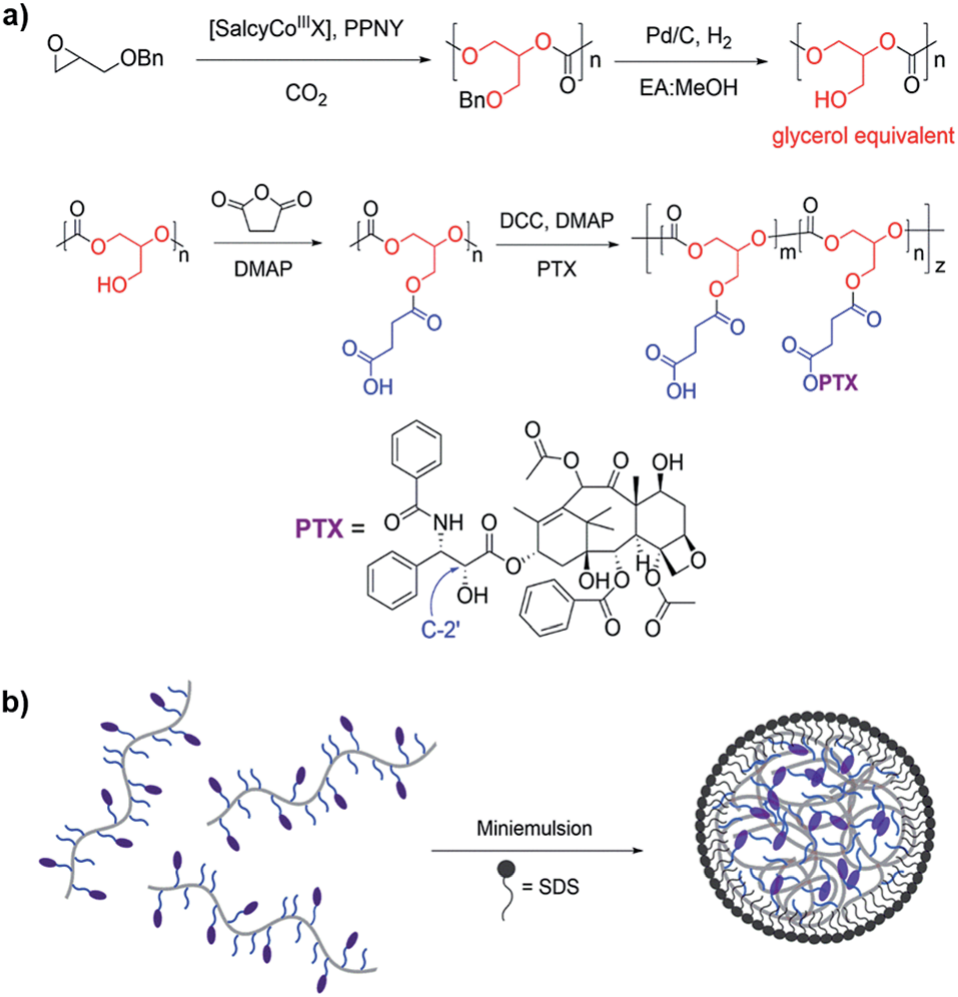
Synthesis and formulation of PGC–PTX NPs. (a) PGC is synthesized by the copolymerization of benzyl glycidyl ether with CO_2_ followed by debenzylation hydrogenation. PGC is then grafted with succinic acid and PTX to afford PGC–PTX conjugate. (b) PGC–PTX NPs are formulated by emulsifying the polymer in the presence of sodium dodecyl sulfate (SDS).

PGC is synthesized *via* the alternating copolymerization of epoxide and carbon dioxide, followed by high pressure hydrogenolysis to remove the benzyl group, as previously described (Fig. 1a).^33^ This metal catalyzed copolymerization reaction is versatile, efficient, and amenable to a large number of epoxide monomers.^39^–^41^ PGC is then treated with succinic anhydride and 4-dimethylaminopyridine (DMAP) to give poly(1,2-glycerol carbonate)-*graft*-succinic acid (PGC-*g*-SA). Standard coupling chemistry using *N*,*N*′-dicyclohexylcarbodiimide (DCC) and DMAP with PTX affords PGC–PTX with high PTX loadings of up to 70 mol%, or 74 wt%. PTX is linked to the polymer backbone *via* hydrolysable ester linkages. PTX incorporation in molar ratio is determined *via*^1^H NMR by integrating the peaks that correspond to the methine proton on the polymer backbone and the C2′ proton on the PTX side chain. To elucidate the site of PTX functionalization, PTX and SA were coupled using DMAP, and the PTX–SA conjugate was isolated by flash column chromatography. Using ^1^H NMR, we determined that PTX binds SA at its C2′-OH as indicated by the downfield chemical shift of the CH proton on C2′ from 4.79 to 5.51 ppm (Fig. S2†). Conjugating PTX at the C2′-OH, which is critical for its activity,^42^,^43^ confers control over the biological activity of the conjugate by necessitating the additional cleavage of PTX into its active form. To study the effect of PTX loading on NP behavior, we focus our evaluation on PGC–PTX with 34, 39, and 43 mol% PTX incorporation (*i.e.*, 34, 39, and 43% PGC–PTX; 58, 61, and 64 wt% PTX respectively) as these polymers are reproducibly synthesized with high PTX incorporation efficiencies of >80%, and form monodisperse NPs of equal size. By size exclusion chromatography, the PGC–PTX constructs used are 9–13 kDa, with polydispersity indices (PDIs) between 1.3 and 1.6 (Table S1†).

**Fig. 2:**
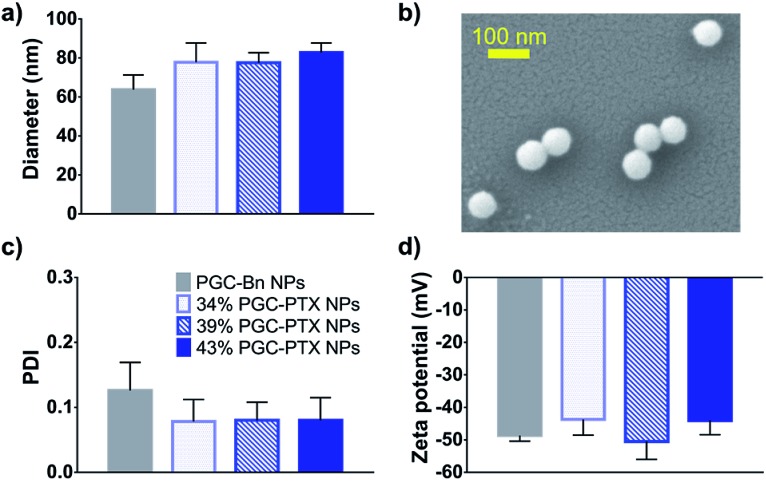
Characterization of PGC–PTX NPs. (a) DLS size measurements of drug free PGC–Bn NPs and PGC–PTX NPs with varying PTX loadings in mol%. (b) SEM micrograph of PGC–PTX NPs. (c) Polydispersity indices and (d) zeta potentials of PGC–Bn and PGC–PTX NPs as measured by DLS. Data is presented as mean ± standard deviation of 3 independently formulated NP batches per group.

It has been previously demonstrated that sub-100 nm particles exhibit effective tumor tissue penetration and retention, while sub-50 nm NPs exhibit poor tumor retention.^7^,^44^,^45^ Therefore, a diameter of 50–100 nm was targeted for the PGC–PTX nanocarriers. PGC–PTX NPs are prepared using a miniemulsion synthesis procedure in which the surfactant, sodium dodecyl sulfate (SDS), is dissolved in phosphate buffer and added to a polymer–dichloromethane solution at a 1 : 5 surfactant : polymer mass ratio (Fig. 1b). The mixture is then emulsified under an argon blanket *via* ultrasonication, and the colloid is purified by dialysis. Dynamic light scattering (DLS) and scanning electron microscopy (SEM) indicate the formation of monodisperse sub-100 nm NPs (Fig. 2a and b). PGC–PTX NP size, PDI, and zeta potential (Fig. 2a, c and d) do not vary with PTX loading. However, drug-free NPs composed of poly(benzyl 1,2-glycerol carbonate) (PGC–Bn), the PGC polymer prior to deprotection, exhibit slightly smaller diameters (63.7 ± 7.6 nm) and greater dispersity (0.126 ± 0.043). The 34, 39, and 43% PGC–PTX NPs possess average diameters of 77.9 ± 9.8 nm, 77.5 ± 5.2 nm, and 82.9 ± 4.8 nm, and PDIs of 0.078 ± 0.034, 0.080 ± 0.028, and 0.080 ± 0.035, respectively. All NPs exhibit similar zeta potentials between –43 and –51 mV due to the negative charge of the SDS surface coating. Using current formulation parameters, NP solutions with markedly high PTX concentrations >15 mg mL^–1^ are prepared, compared to PTX aqueous solubility of 0.3 μg mL^–1^.^46^ The high concentrations of PTX achieved using PGC–PTX NPs thus eliminate the need for additional solubilizing agents such as C/E, while simultaneously reducing carrier material due to high PTX incorporation.

**Fig. 3:**
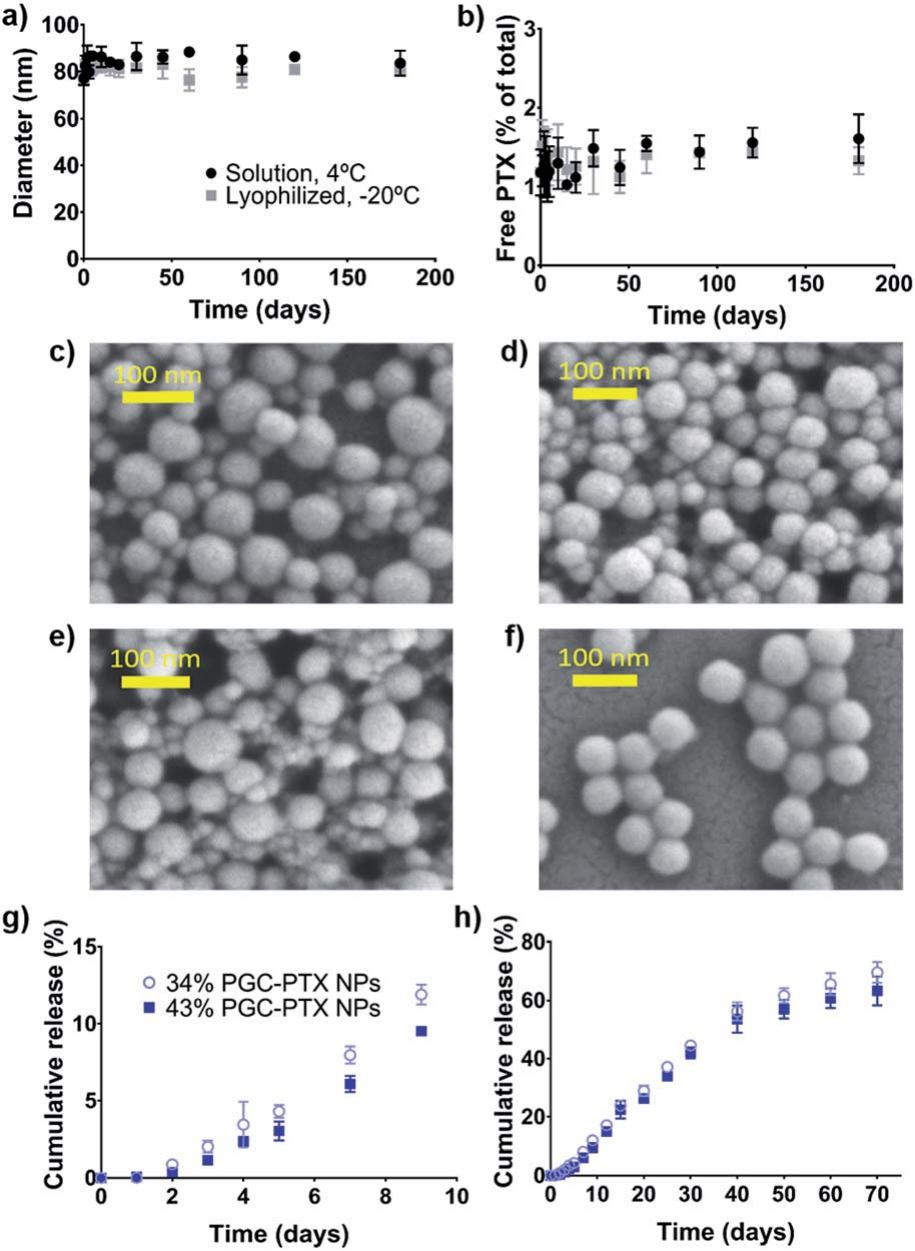
PGC–PTX NP storage stability and release kinetics. PGC–PTX NPs were stored in solution at 4 °C or lyophilized, stored at –20 °C, and resuspended at given time-points. (a) NP diameter and (b) free/unconjugated PTX content were evaluated over the course of 6 months of storage. SEM micrographs of NPs stored as (c, e) a solution or as (d, f) a lyophilized powder after (c, d) 90 and (e, f) 180 days of storage. PTX release kinetics from 34% and 43% PGC–PTX NPs at 37 °C in 0.3% SDS pH 7.4 phosphate buffer at (g) early time-points (<10 days) and over (h) 70 days. All experiments were performed in triplicate, with data presented as mean ± standard deviation.

Among the challenges impeding the translation of nanomedicines into clinical practice are batch-to-batch variation, high dispersity, and poor storage stability.^47^ Having demonstrated that the PGC–PTX NP formulation is robust and reproducible, and that PGC–PTX NPs exhibit narrow dispersities, we next evaluated NP storage stability. The 39% PGC–PTX NPs were used for analysis as a representative population, as this formulation has the median drug loading among the NPs evaluated. The NPs were stored in solution at 4 °C, or lyophilized and stored at –20 °C, without the addition of any stabilizing agents. At later time-points, lyophilized NPs were resuspended in phosphate buffer and both sets of NPs were evaluated for NP size as well as unconjugated PTX content *via* DLS and high performance liquid chromatography (HPLC), respectively. Over the course of 180 days, PGC–PTX NPs maintain a stable diameter of 77–88 nm (Fig. 3a), and free/unconjugated PTX content remains constant at 1–2% of total PTX loading (Fig. 3b). Additionally, resuspended NPs do not differ in size or free PTX content from NPs stored in solution. SEM micrographs confirm the presence of stable NPs after 90 and 180 days of storage (Fig. 3c–f). Taken together, these results demonstrate that PGC–PTX NP physicochemical characteristics are not altered under storage conditions for up to 6 months.

To characterize PTX release kinetics as well as the effect of drug loading, 34% and 43% PGC–PTX NPs were incubated in pH 7.4 phosphate buffer for 70 days at 37 °C (Fig. 3g and h). At given time-points, samples were withdrawn from the release media and free PTX content was determined *via* liquid chromatography-mass spectrometry (LC-MS). At early time-points (<10 days), PTX release is slightly accelerated in 34% PGC–PTX NPs compared to 43% PGC–PTX NPs (Fig. 3g). At later time-points (>10 days), PTX cumulative release is comparable for both formulations (Fig. 3h). An initial burst release is not observed, consistent with release being dependent on both diffusion and cleavage of the drug from the polymer backbone. Furthermore, PGC–PTX NPs exhibit controlled and sustained drug release kinetics, with 70% and 63% cumulative PTX release after 70 days for 34% and 43% PGC–PTX NPs, respectively.

**Fig. 4:**
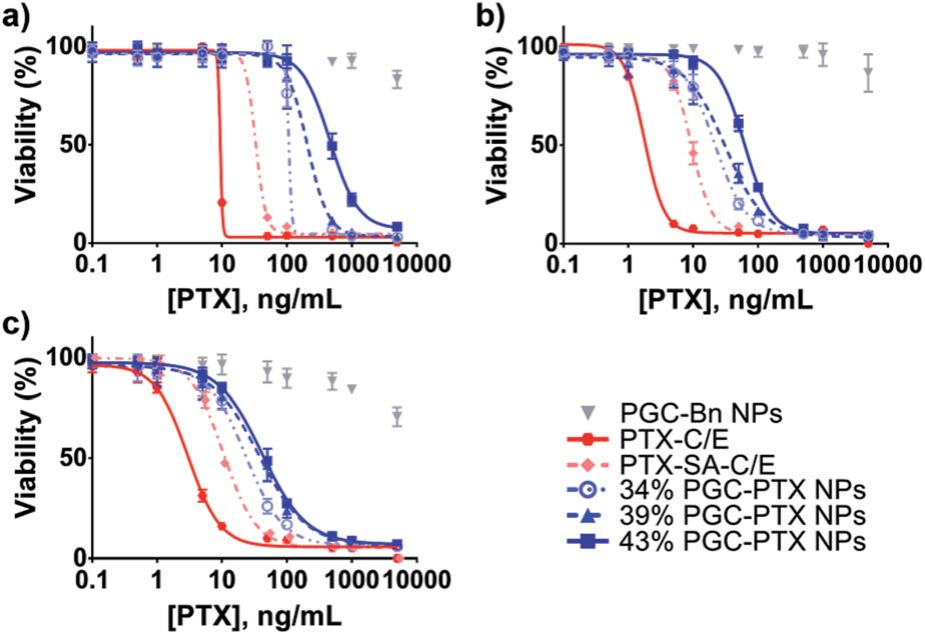
Activity of PGC–PTX NPs against cancer cells *in vitro*. (a) MSTO-211H, (b) A549, and (c) PANC-1 cells were treated with PGC–PTX NPs with varying PTX loadings in mol%, PTX-C/E, PTX–SA-C/E, or drug-free PGC–Bn NPs (given at equivalent PGC backbone concentrations to 34% PGC–PTX NPs). Cell viability was assessed after 5 days of treatment. All experiments were performed in triplicate, with data presented as mean ± standard deviation.

*In vitro* NP cytotoxicity was evaluated after 5 days of treatment in several human cancer cell lines: MSTO-211H mesothelioma cancer cells (Fig. 4a), A549 lung cancer cells (Fig. 4b), and PANC-1 pancreatic cancer cells (Fig. 4c). By conjugating PTX to SA at the C2′-OH position critical for PTX activity, we reduce the *in vitro* potency of PTX–SA-C/E relative to PTX-C/E by requiring the additional cleavage of PTX into its active form. Similarly, due to the continuous and sustained release of PTX from the NP formulations, PGC–PTX NPs exhibit lower *in vitro* potency compared to PTX-C/E (Fig. 4 & Table S2†). A robust correlation between the 50% inhibitory concentration (IC_50_) and drug content suggests that PTX release rates decrease as polymer drug content is increased. This result is in agreement with the measured release kinetics, which indicate that at early time-points (<10 days), drug release is slightly accelerated in 34% PGC–PTX NPs relative to 43% PGC–PTX NPs. Due to the hydrophobic nature of PTX itself, PGC–PTX NPs with higher PTX loadings may exhibit reduced cleavage and release rates owing to less water penetration into the polymer core. Additionally, the increase in molecular weight resulting from higher drug incorporation may result in more compact polymer aggregation in the NP core, further contributing to reduced PTX release. These findings are consistent with those reported by others investigating PTX conjugate polymeric nanoassemblies.^19^,^30^–^32^ Cells treated with drug-free PGC–Bn NPs at equivalent PGC backbone concentrations to 34% PGC–PTX NPs exhibit minimal cell death. However, the reduction in cell viability at high concentrations of PGC–Bn NPs is potentially due to the toxicity of the SDS surface coating (Fig. S7†). Nonetheless, the maximum concentration of SDS used in the treatment of cells *in vitro* is 1.8 μg mL^–1^, which is below the range of previously reported IC_50_ values (43–127 μg mL^–1^).^48^–^50^

**Fig. 5:**
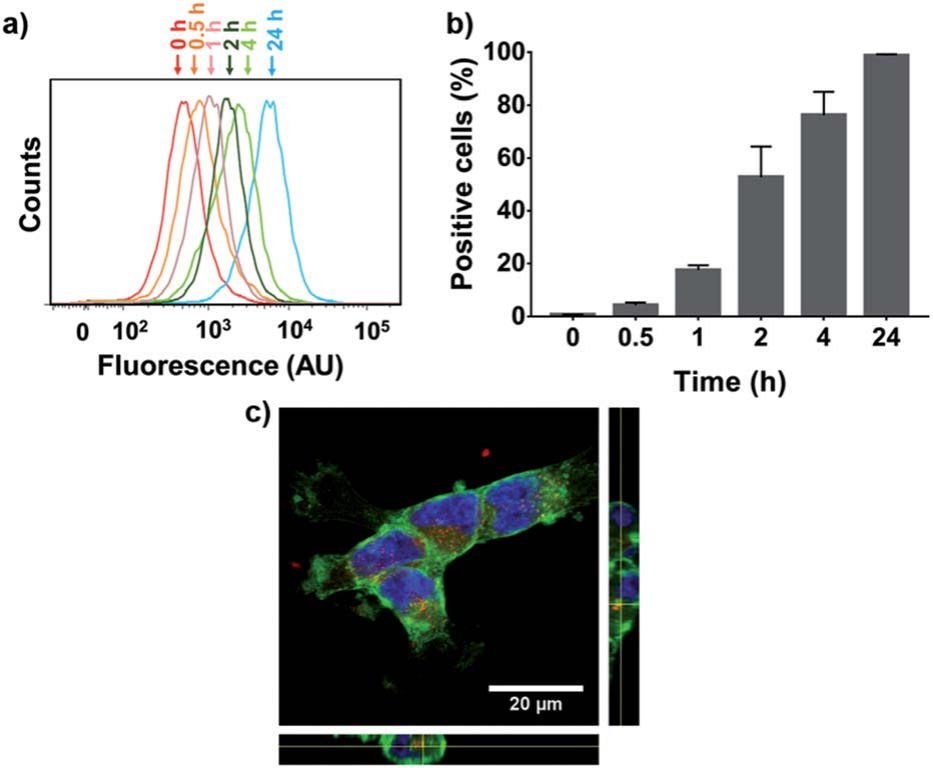
Cellular internalization of PGC–PTX-Rho NPs. Uptake of PGC–PTX-Rho NPs was measured over time in MSTO-211H cells using flow cytometry. (a) A sample set of histograms shows increased fluorescence intensity of the cell population over time. (b) Positive cells are defined as cells which exhibit higher fluorescence than 99% of the control/untreated population. Data presents mean ± standard deviation of three individual experiments. (c) Laser-scanning confocal microscope image of MSTO-211H cells after 24 hours of incubation with PGC–PTX-Rho NPs. Image presented is the 2-D projection of a 3-D, 6 μm *z*-stack. Orthogonal views are shown on the periphery. Cell membranes are visualized in green, nuclei in blue, and NPs in red.

To evaluate NP cellular internalization, a fluorescent rhodamine-labeled PGC–PTX polymer (PGC–PTX-Rho; 10 mol% Rho, 40 mol% PTX) was synthesized by first reacting PGC with rhodamine B isothiocyanate and proceeding with the reaction as previously described to conjugate SA and PTX. PGC–PTX-Rho NPs are formulated in the same manner as PGC–PTX NPs and are physically similar, with a diameter of 78.8 ± 8.0 nm, a PDI of 0.101 ± 0.059, and a zeta potential of –43 ± 8 mV. The maximum dose of PGC–PTX-Rho NPs that can be administered without inducing cell death was determined by evaluating MSTO-211H cell viability 24 h after treatment with 40% PGC–PTX NPs. Given that cell viability exceeds 95% at PTX doses ≤100 ng mL^–1^ (Fig. S8†), MSTO-211H cells were treated with PGC–PTX-Rho NPs at a dose of 100 ng mL^–1^ PTX, and cellular internalization kinetics were assessed *via* flow cytometry at various time-points after treatment (Fig. 5a and b). The increase in fluorescence intensity over time indicates an increase in the portion of the cell population which internalized NPs (Fig. 5a). To quantitate internalization, positive cells were defined as cells which exhibit higher fluorescence than 99% of the control/untreated population (Fig. 5b). PGC–PTX Rho NP internalization occurs rapidly, with >75% of the cell population taking up NPs after 4 h of treatment, and nearly all cells exhibiting NP internalization following 24 h of treatment. PGC–PTX-Rho NP internalization, rather than cell surface adhesion, was confirmed after 24 h of treatment *via* confocal microscopy (Fig. 5c). These results are in agreement with previous studies reporting the internalization of negatively charged NPs.^51^–^54^

Since PGC–PTX NPs contain a substantial quantity of PTX but release their payload gradually and continuously, we hypothesized that PGC–PTX NPs can be safely administered at significantly higher PTX doses than standard PTX-C/E. For reference, the maximum tolerated dose (MTD) of PTX-C/E in mice by IP administration is reported between 13–50 mg kg^–1^, while the median lethal dose (LD_50_) is 128 mg kg^–1^.^55^–^58^ To test this hypothesis, an approved pilot trial was constructed with IACUC guidance, and healthy mice were given 34% PGC–PTX NPs *via* a single IP injection at a dose of 140 mg kg^–1^ PTX. The 34% PGC–PTX NP formulation was chosen for *in vivo* evaluation given its superior *in vitro* efficacy. After treatment with 34% PGC–PTX NPs, mice do not exhibit any signs of acute or chronic toxicity and maintain healthy body weight (Fig. S9a†). All of the mice were euthanized 120 days after treatment, and tissue was harvested for histological analysis to evaluate organ toxicity. Histological evaluation of major organs confirms that the treatment is well tolerated compared to untreated controls (Fig. S9b–m†).

Encouraged by these results, we evaluated the efficacy of PGC–PTX NPs as a single dose in murine models of peritoneal mesothelioma. Mortality in patients with peritoneal mesothelioma commonly results from local disease progression with median survivals of only 4–12 months post-diagnosis.^51^,^59^ Although long-term regional chemotherapy has demonstrated improved outcomes in patients with peritoneal carcinomatosis, its implementation is limited by catheter-related toxicities and intolerance.^5^,^6^ Thus, the optimization of long-term IP chemotherapeutic dosing continues to be of clinical interest. We hypothesized that a single, high-dose PGC–PTX NP IP injection will leverage the utility of the IP route of administration, while exhibiting equivalent efficacy to a multi-dose PTX treatment regimen due to its sustained release profile.

**Fig. 6:**
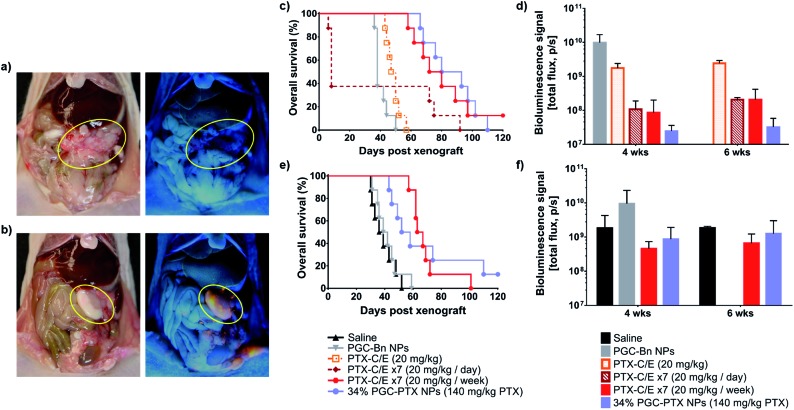
*In vivo* peritoneal distribution and efficacy of PGC–PTX NPs as a single dose. Three weeks after IP tumor inoculation, mice received either (a) rhodamine or (b) PGC–PTX-Rho NPs at equivalent rhodamine doses. Three days after injection, the peritoneum was assessed under (left) ambient and (right) ultraviolet light. The largest IP tumors are circled in yellow in each image. PGC–PTX-Rho NPs are visualized primarily in areas of tumor under ultraviolet light. PGC–PTX NP efficacy was evaluated in a prevention of peritoneal mesothelioma establishment model in which animals (*n* = 8/group) received MSTO-211H-luc cells followed by same-day treatment. (c) Cumulative survival and (d) tumor burden of animals treated with PGC–Bn NPs, 20 mg kg^–1^ PTX-C/E, daily 20 mg kg^–1^ PTX-C/E for 7 days, weekly 20 mg kg^–1^ PTX-C/E for 7 weeks, or 140 mg kg^–1^ PTX *via* 34% PGC–PTX NPs. PGC–PTX NP efficacy was also evaluated in the treatment of established peritoneal mesothelioma. (e) Cumulative survival and (f) tumor burden of animals treated with saline, PGC–Bn NPs, weekly 20 mg kg^–1^ PTX-C/E for 7 weeks, or 140 mg kg^–1^ PTX *via* 34% PGC–PTX NPs. Bioluminescence data is presented as the mean ± standard deviation for 3 randomly assigned animals per group.

First, to determine NP distribution within the peritoneal cavity, mice received an IP injection of either PGC–PTX-Rho NPs or rhodamine in saline three weeks after IP MSTO-211H tumor inoculation. Three days after injection, animals were euthanized, and the peritoneum was assessed under ambient and ultraviolet light. By gross inspection, NPs are visualized primarily in areas of tumor, suggesting that PGC–PTX NPs preferentially localize to IP tumors following local administration (Fig. 6a and b). This observation is consistent with previous reports on the localization of negatively charged NPs to peritoneal tumors following IP administration, and may be attributed to the increased metabolic activity and more rapid NP internalization of tumor cells compared to healthy cells.^51^,^59^–^61^ Quantitative and mechanistic studies exploring this phenomenon are underway, including the evaluation of the distribution and localization of fluorescently-labeled, non-drug loaded PGC NPs.

We next evaluated the efficacy of PGC–PTX NPs in preventing the establishment of mesothelioma locally in the peritoneum. Immediately following IP tumor inoculation with luciferase-expressing MSTO-211H (MSTO-211H-luc) cells, animals received one of several treatments *via* separate IP injection. Treatment groups included a single dose of 140 mg kg^–1^ PTX as 34% PGC–PTX NPs, PGC–Bn NPs (polymer backbone control), 20 mg kg^–1^ PTX-C/E (single PTX dose control), 7 × 20 mg kg^–1^ daily PTX-C/E (total dose control), and 7 × 20 mg kg^–1^ weekly PTX-C/E (total dose control). Given the known lethality of a bolus dose of 140 mg kg^–1^ PTX-C/E, this positive control could not be justified, and therefore, equivalent PTX dose comparisons were achieved with seven injections of 20 mg kg^–1^ PTX-C/E at daily or weekly dosing intervals. Nonetheless, animals that received 7× daily doses of 20 mg kg^–1^ PTX-C/E exhibit a 63% acute mortality rate due to PTX-C/E toxicity (Fig. 6c). Conversely, when the equivalent PTX-C/E dose is administered over seven weeks, acute toxicity is decreased and animals exhibit comparable survival to those receiving a single dose of 140 mg kg^–1^ PTX *via* PGC–PTX NPs (median survivals of 76 and 86.5 days, respectively; *p* = 0.77). Similarly, at 4 and 6 weeks after tumor inoculation, PGC–PTX NP treated animals exhibit comparable tumor burden to animals treated with a weekly PTX-C/E multi-dose regimen (Fig. 6d). Therefore, a single high dose of PTX can be safely administered *via* PGC–PTX NPs without the risk of C/E hypersensitivity or other toxicities, and with comparable efficacy to PTX-C/E administered as a multi-dose regimen over the course of weeks.

To determine whether these findings are replicated in an established model of peritoneal mesothelioma, animals were treated one week after tumor inoculation with either saline, PGC–Bn NPs (polymer backbone control), or 140 mg kg^–1^ PTX administered as a single dose of 34% PGC–PTX NPs or as 7 × 20 mg kg^–1^ weekly PTX-C/E (total dose control). Consistent with our previous findings, animals treated with PGC–PTX NPs or 7× weekly PTX-C/E exhibit similar tumor burden at 4 and 6 weeks after tumor inoculation, and overall survival is comparable between PGC–PTX NP and 7 × 20 mg kg^–1^ weekly PTX-C/E groups (median survivals of 55 and 65 days, respectively; *p* = 0.80) (Fig. 6e and f). Due to their improved safety profile and sustained PTX release kinetics, PGC–PTX NPs can therefore supplement chemotherapeutic dosing as a safe and facile replacement for multiple PTX-C/E administrations.

## Conclusions

The conjugation of small molecule anticancer agents to polymeric carriers is a promising approach for overcoming the clinical limitations of cytotoxic chemotherapeutics. The progression of PTX poliglumex into clinical evaluation highlights the potential of this approach to improve patient outcomes. Nonetheless, several key features distinguish PGC–PTX NPs from PTX poliglumex. First, PGC–PTX NPs are a colloidal drug delivery system, whereas PTX poliglumex is a linear, water-soluble polymer–drug conjugate. The formulation of the hydrophobic PGC–PTX polymer into colloidal NPs affords high drug loadings of up to 74 wt%. Since PTX poliglumex relies on the improved aqueous solubility afforded by the poly-l-glutamic acid backbone, PTX loading is limited to 37 wt%.^26^ Additionally, the hydrolytic release of PTX from PTX poliglumex occurs at a rate of approximately 12% per day, with most of the drug being released within 8 days.^62^ On the contrary, PGC–PTX nanocarriers exhibit controlled and sustained drug release over an extended period of 70 days, with <15% of the drug being released over an 8 day period. Although both systems employ an ester linkage between the drug and the carrier, the significant difference in release kinetics is likely due to the sequestration of PTX within the hydrophobic core of the PGC–PTX NP, which is not achieved in the water-soluble PTX poliglumex. The extended drug release exhibited by PGC–PTX NPs enables their use as a single, high-dose replacement for multi-dose PTX treatment regimens, especially for the treatment of peritoneal cancers, in which the implementation of local, multi-dose treatment regimens is challenging. While a previously published report illustrates the utility of single, high-dose PTX poliglumex in the local treatment of peritoneal cancers relative to multi-dose PTX regimens, PTX-treated animals received significantly lower total drug (15–60 mg kg^–1^ PTX given over 3 administrations) compared to animals treated with PTX poliglumex (140–200 mg kg^–1^ PTX equivalent), confounding a direct comparison between the two treatment groups.^63^

In summary, our work presents the rational design of a biodegradable polymeric nanocarrier in which PTX is incorporated at high, controlled loadings of up to 74 wt% and in which PTX aqueous concentrations exceed 15 mg mL^–1^. Importantly, PGC presents a generalizable platform for the controlled conjugation and delivery of other therapeutic or imaging agents. The PGC–PTX NP formulation is robust, producing monodisperse sub-100 nm NPs with long-term storage stability and sustained PTX release kinetics. Due to the controlled and sustained release of PTX, PGC–PTX NPs are safely administered at doses exceeding the LD_50_ of PTX-C/E. *In vivo*, PGC–PTX NPs preferentially accumulate in IP tumors after local administration, similar to other negatively charged NPs.^60^ Furthermore, a single dose of PGC–PTX NPs exhibits an equivalent oncologic effect to seven weekly doses of PTX-C/E, rendering PGC–PTX NPs an ideal platform for increasing patient compliance, reducing costs associated with visits, and eliminating the use of C/E and its related toxicities. Notably, PGC–PTX NPs present a unique drug delivery system for the improvement and optimization of chemotherapeutic dosing regimens by enabling the facile implementation of a high dose, sustained release treatment platform.

## Conflicts of interest

There are no conflicts to declare.

## Supplementary Material

Supplementary information
