# Supplementary material for: Synthesis of poly(1,2-glycerol carbonate)–paclitaxel conjugates and their utility as a single high-dose replacement for multi-dose treatment regimens in peritoneal cancer
Source: Chem Sci. 2017 Oct 20;8(12):8443–50. doi: 10.1039/c7sc03501b (PMC5863611; doi:10.1039/c7sc03501b)
Supplement: Supplementary file 1 [file SC-008-C7SC03501B-s001.pdf]

Electronic Supplementary Material (ESI) for Chemical Science.
This journal is © The Royal Society of Chemistry 2017

Supplementary Information

Synthesis of poly(1,2-glycerol carbonate)-paclitaxel conjugates and their utility as a single high-dose replacement for multi-dose treatment regimens in peritoneal cancer

Iriny Ekladious,[a] Rong Liu,[b] Heng Zhang,[a] Daniel H. Foil,[c] Daniel A. Todd,[c] Tyler N. Graf,[c] Robert F. Padera,[d] Nicholas H. Oberlies,[c] Yolonda L. Colson*[b] and Mark W. Grinstaff*[a]

[a.] Departments of Biomedical Engineering and Chemistry, Boston University, Boston, MA 02215, USA. Email: mgrin@bu.edu

[b.] Department of Surgery, Brigham and Women's Hospital, Boston, MA 02215, USA. Email: ycolson@bwh.harvard.edu

[c.] Department of Chemistry and Biochemistry, University of North Carolina at Greensboro, Greensboro, NC 27402, USA

[d.] Department of Pathology, Brigham and Women's Hospital, Boston, MA 02215, USA

Experimental procedures

General information:

All manipulations involving air- and water-sensitive compounds were carried out in a glovebox. Benzyl glycidyl ether was refluxed over CaH_2, and fractionally distilled under nitrogen atmosphere prior to use. Carbon dioxide (99.995%, research grade) was purchased from Airgas (Dorchester, MA) and used as received. Pd/C (10%) (wetted with *ca.* 50% water) were purchased from Strem Chemicals (Newburyport, MA) and used as received. ^1H and ^{13}C NMR spectra were recorded on a Varian 500 MHz type (^1H, 500MHz; ^{13}C, 125 MHz) spectrometer (Varian, Inc.; Palo Alto, CA). Their peak frequencies were referenced against the solvent, chloroform-*d* at δ 7.24 for ^1H NMR and δ 77.23 ppm for ^{13}C NMR; and dimethyl sulfoxide-(DMSO-)*d6* at δ 2.50 for ^1H NMR and δ 39.52 for ^{13}C NMR. Molecular weights were determined via size exclusion chromatography calibrated against polystyrene standards using tetrahydrofuran (THF) as the eluent at a flow rate of 1.0 mL/min through a Styragel HR 4E 7.8 x 300 mm THF column (Waters Corporation; Milford, MA) with a Wyatt Technology Optilab DSP Interferometric Refractometer (Santa Barbara, CA).

Synthetic procedure:

Synthesis of PGC-*g*-SA. Poly(1,2-glycerol carbonate) (PGC) was synthesized via the alternating copolymerization of epoxide and CO_2 followed by high pressure hydrogenolysis to remove the benzyl group, as previously described[1]. PGC-PTX conjugate was synthesized by first reacting PGC with succinic anhydride to provide poly(1,2-glycerol carbonate)-*graft*-succinic acid (PGC-*g*-SA), which possesses a carboxylic acid on each repeating unit. PGC (100 mg, 0.85 mmol, 1.0 eq), succinic anhydride (93 mg, 0.93 mmol, 1.1 eq.), and 4-dimethylaminopyridine (DMAP) (5.2 mg, 0.042 mmol, 0.05 eq.) were added into a 5 mL round bottom flask. Dimethylformamide (DMF; 1 mL) was then added to dissolve the solid, and the reaction was stirred at room temperature overnight. The reaction mixture was then added dropwise into diethyl ether. After centrifugation, the upper layer was decanted and the solid was re-dissolved with an ethyl acetate/methanol mixture (3:2). The solution was then added dropwise into diethyl ether for a second precipitation, and the mixture was centrifuged again to collect the polymer. The isolated polymer was dried under vacuum overnight, and the polymer was isolated as a white foam. ^1H NMR (500 MHz, DMSO-*d6*, ppm): δ 5.02-5.23 (br, 1H), 4.11-4.46 (br, 4H), 2.38-2.60 (br, 4H); ^{13}C NMR (125 MHz, DMSO-*d6*, ppm): δ 166.0, 153.6, 134.8, 128.8, 128.4, 73.5, 68.0, 66.2, 53.7 (see Fig. S1).

Synthesis of PTX-SA. PTX (100 mg, 0.117 mmol, 1.0 eq.), succinic anhydride (11.1 mg, 0.111 mmol, 1.05 eq.), and DMAP (0.71 mg, 0.0059 mmol, 0.05 eq.) were added into a 5 mL round bottom flask. DMF (1 mL) was added to dissolve the solid, and the reaction was stirred at room

temperature overnight. PTX-SA was then isolated by flash column chromatography, yield: 95 mg, 85%. ^{1}H NMR analysis revealed that the PTX C2'-OH is the only site that is active under the reaction conditions described (Fig. S2). ^{1}H NMR (500 MHz, chloroform-*d*, ppm): δ 1.13 (s, 3H, (C-16)-C*H*3), 1.25-1.40 (s, 3H, (C-17)-C*H*3), 1.50-1.70 (m, 3H, (C-19)-C*H*3), 1.82-1.95 (m, 5H, (C-6)-C*H*, 1-O*H* and (C-18)-C*H3*), 2.15 (m, 1H, (C-14)-C*H*), 2.23 (s, 3H, 10-OAc), 2.34-2.62 (m, 10H, 4-OAc, (C-6)-C*H*, (C-14)-C*H*, 7-O*H*, HOOCCH_2CH_2CO-), 3.81 (d, 1H, (C-3)-C*H*), 4.20 (d, 1H, (C-20)-C*H*), 4.31 (d, 1H, (C-20)-C*H*), 4.46 (m, 1H, (C-7)-C*H*), 4.94 (dd, 1H, (C-5)-C*H*), 5.51 (d, 1H, (C-2')-C*H*), 5.68 (d, 1H, (C-2)-C*H*), 5.95 (dd, 1H, (C-3')-C*H*), 6.23-6.29 (m, 2H, (C-10)-C*H* and (C-13)-C*H*), 6.85 (d, 1H, 3'-N*H*), 7.34-7.64 (m, 11H, Ph*H*), 7.73 (d, 2H, Ph*H*), 8.14 (d, 2H, Ph*H*); ^{13}C NMR (125 MHz, chloroform-*d*, ppm): δ 9.6, 14.9, 24.2, 28.4, 35.6, 43.2, 45.6, 51.1, 52.8, 58.5, 71.8, 72.2, 73.9, 75.1, 75.6, 76.5, 79.2, 81.1, 84.5, 126.5, 127.1, 128.5, 128.8, 129.1, 129.2, 130.3, 132.1, 132.8, 133.7, 137.0, 142.8, 167.1, 168.1, 169.8, 171.3, 172.5, 175.5, 203.9.

Synthesis of PGC-PTX. PGC-*g*-SA was conjugated with PTX under standard coupling conditions to afford PGC-*g*-SA-PTX conjugate (PGC-PTX). As an example, to synthesize PGC-PTX with 34 mol% PTX loading, PGC-*g*-SA (100 mg, 0.459 mmol, 1.0 eq.), PTX (157 mg, 0.183 mmol, 0.4 eq.), and N,N'-dicyclohexylcarbodiimide (DCC) (42 mg, 0.202 mmol, 0.44 eq.) were added into a 5 mL round bottom flask. DMF (1 mL) was added to dissolve the solid and the reaction was stirred at room temperature overnight. The reaction mixture was then filtered using a 0.2 µm Millex-GN nylon syringe filter (EMD Millipore; Billerica, MA) to remove precipitated dicyclohexylurea (DCU). The filtrate was added dropwise into diethyl ether. After centrifugation, the upper layer was decanted and the solid was re-dissolved with dichloromethane (DCM). The solution was then added dropwise into diethyl ether for a second precipitation and the solution was centrifuged again to collect the conjugate. The conjugate was isolated as a white powder (172 mg, 75% from PGC-SA). The percentage of PTX loading was determined via ^{1}H NMR by integrating the peaks that correspond to the methine proton on the polymer backbone and the C2' proton on the PTX side chain (Fig. S3).

Synthesis of PGC-PTX-Rho. PGC (100 mg, 0.85 mmol, 1.0 eq), rhodamine B isothiocyanate (36.4 mg, 0.068 mmol, 0.08 eq), and DMF (1 mL) were added into a 5 mL round bottom flask. The reaction mixture was stirred at room temperature for 6 hours. Succinic anhydride (93 mg, 0.93 mmol, 1.1 eq.) and DMAP (5.2 mg, 0.042 mmol, 0.05 eq.) were then added, and the reaction was stirred at room temperature overnight. The reaction mixture was then added dropwise into diethyl ether. After centrifugation, the upper layer was decanted and the solid was re-dissolved with an ethyl acetate/methanol mixture (3:2). The solution was then added dropwise into diethyl ether for a second precipitation, and the mixture was centrifuged again to collect the polymer. The isolated polymer was allowed to dry on vacuum overnight, and the polymer was isolated as red foam. Subsequently, PTX (157 mg, 0.183 mmol, 0.4 eq.), DCC (42 mg, 0.202

mmol, 0.44 eq.), and DMF (1 mL) were added to the polymer, and the solution was stirred at room temperature overnight. The reaction mixture was then filtered using a 0.2 µm Millex-GN nylon syringe filter (EMD Millipore; Billerica, MA) to remove precipitated DCU. The filtrate was then added dropwise into diethyl ether. After centrifugation, the upper layer was decanted and the solid was re-dissolved with DCM. The solution was then added dropwise into diethyl ether for a second precipitation and the solution was centrifuged again to collect the PGC-PTX-Rho conjugate. PGC-PTX-Rho was isolated as a dark red powder (201 mg, 55% from PGC). The percentage of PTX drug loading and rhodamine conjugation were determined via ^{1}H NMR (Fig. S4).

NP synthesis

NPs were prepared using a miniemulsion synthesis procedure. Briefly, 10-50 mg of polymer were dissolved in 0.5 mL DCM and added to a 2 mL solution of sodium dodecyl sulfate (SDS) in pH 7.4 10 mM phosphate buffer at a 1:5 SDS:polymer mass ratio. The mixture was then emulsified under an argon blanket using a Sonics Vibra-Cell VCX-600 Ultrasonic Processor (Sonics & Materials; Newtown, CT). Following sonication, the NP suspension was subjected to stirring under argon for 2 hours, followed by stirring under air overnight to allow for the evaporation of remaining solvent. The resulting NP suspension was dialyzed for 24 hours against 1 L of 5 mM pH 7.4 phosphate buffer. The dialysis buffer was exchanged with fresh buffer after 10 hours of dialysis. PGC-PTX-Rho NPs used for *in vivo* peritoneal distribution studies were formulated using 1% (wt/wt) PGC-PTX-Rho polymer (10 mol% Rho, 41 mol% PTX) in combination with 40 mol% PTX PGC-PTX polymer (40% PGC-PTX). PGC-PTX-Rho NPs used in *in vitro* studies were formulated using PGC-PTX-Rho alone.

Calculation and confirmation of NP PTX loading

NPs were prepared using conjugates of known PTX loading, as determined via ^{1}H NMR by integrating the peaks that correspond to the methine proton on the polymer backbone and the C2’ proton on the conjugated PTX (Fig. S3). PTX incorporation in molar ratio was then converted to mass ratio, and this value was used to calculate NP PTX concentration from a known PGC-PTX NP concentration. To confirm the incorporation of all added polymer into the resultant NP solution, NPs were diluted in acetonitrile to achieve a concentration of 20 µg/mL PTX. The absorbance of the solution at 227 nm was then determined using a Hewlett Packard 8453 UV-Vis Spectrophotometer (The Hewlett-Packard Company; Palo Alto, CA). A set of PGC-PTX polymer standards was used to correlate absorbance to PTX concentration. A linear standard curve was developed (R^2 = 0.999) and used to confirm PTX loading. PTX loading is reported as ± 1 mol%.

NP diameter, PDI, and zeta potential

Dynamic light scattering (DLS) was used to measure particle diameter, PDI, and zeta potential using a Brookhaven 90Plus Particle Size Analyzer (Brookhaven Instruments Corporation; Holtsville, NY). NPs were diluted in DI water in order to achieve a count rate of approximately 150 kilocounts per second. Zeta potentials were measured using the Brookhaven Instruments Corporation ZetaPALS zeta potential analyzer via the Smoluchowski method. Data presented are the averages of number-weighted size distributions ± standard deviation (SD) of three independently prepared samples per NP formulation. Statistical analysis was performed with one-way analysis of variance (ANOVA).

NP visualization via scanning electron microscopy (SEM)

A Zeiss SUPRA 55VP field emission (FE)-SEM (Carl Zeiss Microscopy; Jena, Thuringia, Germany) was used to visualize NPs. Samples were prepared by diluting the NP suspension 1000x in DI water, and dropping a 10 µL aliquot on a silicon wafer. After samples were allowed to air-dry overnight, they were coated with Au/Pd using a Cressington 108 Manual Sputter Coater (Cressington Scientific Instruments; Watford, England, UK).

Storage stability

The 39% PGC-PTX NPs were synthesized and the starting diameters were measured using DLS. The initial free/unconjugated PTX content was determined by reversed-phase high performance liquid chromatography (HPLC) using a 7:3 acetonitrile:water solution as the eluent at a flow rate of 0.5 mL/min through a HxSil C18 4.6 x 250 mm column (Hamilton Robotics; Reno, NV) with a ProStar 335 Diode Array Detector (Varian, Inc; Palo Alto, CA). A set of free PTX standards was used to correlate the integrated area under the peak, at an absorbance of 228 nm, to concentration. Three linear standard curves were developed (0.1 - 5 µg/mL [R^2 = 0.989], 5 – 50 µg/mL [R^2 = 0.997], and 50 – 500 µg/mL [R^2 = 0.999]) and used to determine unknown PTX concentrations. To prepare the NP solutions for HPLC analysis, 30 µL of NP suspension was added to 970 µL acetonitrile, vortexed for 1-2 minutes, and filtered using a 0.2 µm Millex-GN nylon syringe filter (EMD Millipore; Billerica, MA).

Following initial NP characterization, half of the NP solution was stored at 4°C, and the remaining solution was divided into 50 µL aliquots, lyophilized, and stored at -20°C. After 1, 2, 3, 4, 5, 10, 15, 20, 30, 45, 60, 90, 120, or 180 days of storage, lyophilized NPs were resuspended in 70 µL of pH 7.4 10 mM phosphate buffer. At the given time points, both resuspended NPs as well as NPs stored at 4°C were characterized in order to determine NP diameter and free PTX content. After 90 and 180 days of storage, NPs were visualized via SEM. Data represents the average ± SD of N=3/group. Statistical analysis was performed with two-way ANOVA.

PTX release kinetics

Release medium was prepared by adding 0.3% w/w SDS to 10 mM pH 7.4 phosphate buffer following a published procedure to ensure sink conditions[2]. 34% or 44% PGC-PTX NPs (1.98 mg PTX equivalent) were diluted with release medium to achieve a total volume of 10 mL. This solution was then placed into 10,000 molecular weight cutoff dialysis tubing, and the tubing was placed into 290 mL of release buffer stirring at 37°C. At given time-points, 400 µL samples were withdrawn from the release medium and replaced with fresh release buffer. The withdrawn samples were immediately quenched with 800 µL cold acetonitrile, vortexed for 20 seconds, and stored at -20°C. In a separate experiment, PTX degradation kinetics were evaluated by adding 1.98 mg PTX to 300 mL release buffer stirring at 37°C. At given time-points, 400 µL samples were withdrawn from the release medium, immediately quenched with 800 µL cold acetonitrile, vortexed for 20 seconds, and stored at -20°C. PTX content was determined using a Q Exactive Plus hybrid quadrupole-orbitrap mass spectrometer (Thermo Scientific; Waltham, MA) with an electrospray ionization source coupled to an Acquity UPLC system (Waters Corp.; Milford, MA). A 7.5 µL injection of each sample was eluted from an Acquity UPLC HSS C18 2.1 x 50 mm column using a binary solvent gradient consisting of water with 0.1% formic acid and acetonitrile with 0.1% formic acid. The mass spectrometer was operated in positive ionization mode over a scan range of 500-2000 at a resolution of 35,000 with the following settings: spray voltage set at 3700 V, capillary temperature set at 256 °C, sheath gas pressure set to 35, auxiliary gas pressure set to 15, sweep gas set to 2.25, heater temperature set to 412 °C, and S-Lens RF level set to 75. An external standard curve, prepared using 2-fold dilutions of a PTX standard from 1 to 2000 nM in 1:3 release buffer:acetonitrile, was used to determine PTX concentration. Peak area of the extracted-ion chromatogram for m/z 854.3387 ± 5 ppm was plotted against calculated concentrations and subjected to linear regression with a $1/x^2$ weighting. Data represents the average ± SD of N=3/group. Statistical analysis was performed with two-way ANOVA.

A conservative approach was utilized to compensate for PTX degradation (Fig. S6). We assumed that drug released between time-points t_1 and t_2, $\delta M(t_2)$, was released immediately before sample collection at t_2 and was thus not affected by degradation. However, PTX content measured at t_1, $M(t_{1,measured})$, exhibits degradation over the time period (t_2 - t_1), or δ. Therefore at t_2, $M(t_{1,measured})$ has a new value $D_\delta * M(t_{1,measured})$, where D_δ is the known ratio of PTX remaining after δ time as determined from PTX degradation kinetics. Knowing $M(t_{1,measured})$, $M(t_{2,measured})$, and D_δ, we can calculate $\delta M(t_2)$ using the following relationship: $M(t_{2,measured}) = D_\delta * M(t_{1,measured}) + \delta M(t_2)$. Therefore, the corrected cumulative amount of drug released by time-point t_2, $M(t_{2,corrected}) = M(t_{1,corrected}) + \delta M(t_2)$. This approach was used iteratively to calculate corrected PTX release based on measured PTX release and degradation kinetics. The assumption used in this approach provides minimum values for release, and true values may be higher.

Cell culture

MSTO-211H, MSTO-211H-luc (firefly luciferase gene transfected MSTO-211H cells, MSTO-211H/CMMPnlacZ/LucNeo [5/11/04T]), and A549 cells (ATCC; Manassas, VA) were cultured in Roswell Park Memorial Institute (RPMI) medium supplemented with 10% fetal bovine serum (FBS) and 1% penicillin−streptomycin. PANC-1 cells (ATCC) were cultured in Dulbecco's Modified Eagle Medium (DMEM) supplemented with 10% FBS and 1% pencillin-streptomycin. Cells were maintained at 37°C with 5% CO_2 in a humidified environment.

In vitro cytotoxicity

MSTO-211H, PANC-1, and A549 cells were seeded in 96-well plates at densities of 3000, 4000, and 1000 cells/well, respectively, and allowed to adhere for 24 hours. On each plate, blank wells (media only) were defined as 0% viability, and wells with untreated cells were defined as 100% viability. Dilutions of PGC-PTX NPs (34, 39, and 43 mol% PTX; or 58, 61, and 64 wt% PTX, respectively), PTX formulated in Cremophor EL/ethanol (1:1 v/v; PTX-C/E), or PTX-SA-C/E were prepared, and cells were treated with 5000, 1000, 500, 100, 50, 10, 5, 1, 0.5, or 0.1 ng/mL PTX for 5 days. Cells were also treated with poly(benzyl 1,2-glycerol carbonate) (PGC prior to debenzylation; PGC-Bn) NPs as a drug free NP control (administered at equivalent PGC backbone concentrations as 34% PGC-PTX NPs; 2015.22, 403.04, 201.52, 40.30, 20.15, 4.03, 2.02, 0.40, 0.20, 0.04 ng/mL PGC backbone equivalent) for 5 days. MSTO-211H cells were additionally treated with SDS alone (administered at equivalent concentrations to those employed in the 34% PGC-PTX NP formulation: 1813.66, 362.73, 181.37, 36.27, 18.14, 3.63, 1.81, 0.36, 0.18, 0.04 ng/mL SDS) for 5 days. Viability was assessed using the 3-(4,5-dimethylthiazol-2-yl)-5(3-carboxymethonyphenol)-2-(4-sulfophenyl)-2H-tetrazolium (MTS) assay (CellTiter 96 Aqueous One, Promega; Madison, WI). The absorbance at 492 nm was determined using a Beckman Coulter AD 340 plate reader (Beckman Coulter Inc.; Brea, CA). The percent viability was determined by subtracting the average of media blanks and normalizing the absorbance of each well to the average absorbance of the untreated cells. The 50% inhibitory concentrations, or IC_{50} values, were determined by fitting the resultant data to sigmoidal four-parameter logistic curves using GraphPad Prism (GraphPad Software, Inc.; La Jolla, CA). Each curve represents the average of three experiments in which n=6-8/treatment group. To determine the maximum concentration of PGC-PTX-Rho NPs that can be administered within 24 hours without inducing cell death, MSTO-211H cells were seeded in 96-well plates (N=3) at a density of 15,000 cells/well and allowed to adhere for 24 hours. Cells were treated with dilutions of 40% PGC-PTX NPs and viability was assessed after 24 hours using the MTS assay as described previously (n=8/treatment group).

In vitro cellular uptake: flow cytometry

MSTO-211H cells were seeded in 6-well plates at a density of 300,000 cells/well and allowed to adhere for 24 hours. Cells were then incubated with PGC-PTX-Rho NPs at a dose of 100 ng/mL PTX, which was determined to be the maximal concentration of PTX that can be administered as PGC-PTX NPs (40 mol% PTX) without inducing cell death after 24 hours of exposure (> 95% cell viability; Fig. S7). After 0, 0.5, 1, 2, 4, or 24 hours of incubation (N=3/time point), wells were rinsed 3x with 1 mL/well Dulbecco's phosphate buffered saline (PBS) without calcium or magnesium. The cells were detached with 0.5 mL/well trypsin-EDTA (0.25%), and 2 mL media was added to each well to collect the cells. The cells were pelleted via centrifugation at 1000 RPM for 5 minutes, and the supernatant was removed. The cells were then re-suspended in 3 mL 4% formaldehyde and incubated for 15 minutes. Cells were washed by adding 5 mL cold fluorescence-activated cell sorting (FACS) buffer (0.1% sodium azide, 1% BSA in PBS) and re-suspending. The cell suspensions were centrifuged at 1000 RPM for 5 minutes, the supernatant was removed, and the cells were re-suspended in 0.75 mL FACS buffer. Fluorescence was evaluated using a BD LSRII flow cytometer (BD Biosciences; Franklin Lakes, NJ), with 10,000 events (single cells) recorded per sample. FACSDiva (Version 6.2; BD Biosciences) software was used to quantify cells which had internalized NPs (i.e., positive cells). These were defined as cells which exhibited higher fluorescence than 99% of the control/untreated population.

In vitro cellular uptake: confocal microscopy

MSTO-211H cells were seeded in 6-well plates at a density of 200,000 cells/well and allowed to adhere for 24 hours onto 25 mm diameter, # 1.5 thickness, sterile, Poly-L-lysine coated coverslips (Neuvitro Corporation; Vancouver, WA). Cells were then incubated with PGC-PTX-Rho NPs at a dose of 100 ng/mL PTX, as previously described. After 24 hours of incubation, wells were rinsed 2x with 1 mL/well PBS and fixed in 4% formaldehyde for 20 minutes. The coverslips were rinsed 2x with 1 mL/well PBS, and incubated for 8 minutes with 100 μg/mL Concanavalin A-fluorescein conjugate (ThermoFisher Scientific; Waltham, MA) for membrane staining and 3 μg/mL Hoechst 33342 trihydrochloride trihydrate (ThermoFisher Scientific) for nuclear staining. Wells were washed 2x with 1 mL/well PBS, and coverslips were mounted onto glass slides using ProLong Gold Antifade Mountant (ThermoFisher Scientific). Slides were visualized using a Zeiss LSM 710 confocal microscope (Carl Zeiss Microscopy; Jena, Thuringia, Germany). Images were analyzed using ImageJ software (NIH; Bethesda, MD).

Animals used in in vivo studies

Athymic female mice (6-8 week old NU/J; The Jackson Laboratory; Bar Harbor, ME) were housed at the animal facility of Dana Farber Cancer Institute (Boston, MA) under sterile conditions. Animal care and procedures were conducted with Institutional Animal Care and Use Committee approval, in strict compliance with all federal and institutional guidelines for the care and use of laboratory animals.

In vivo NP safety

Three healthy NU/J mice received a 140 mg/kg PTX dose as 34% PGC-PTX NPs via intraperitoneal (IP) injection. Mice were housed with free access to food and water, and were euthanized 120 days after IP injection. Two healthy NU/J mice of the same age served as untreated controls, and were housed for more than two weeks in separate cages at the same animal facility. Tissues including heart, lung, liver, spleen, kidney, and intestine were harvested, fixed with 10% formalin, paraffin embedded, and stained with hematoxylin and eosin (H&E). Pathological evaluation was performed by a licensed pathologist (R.F.P.).

In vivo NP peritoneal distribution

Three weeks after IP tumor inoculation (5x10^6 MSTO-211H-luc cells), animals received an IP 100 uL/10 g injection of either PGC-PTX-Rho NPs (n=3) or rhodamine (n=2) in saline at a concentration of 0.03 mg/mL rhodamine. Three days after injection, animals were sacrificed and digital photographs using automatic exposure were taken using a customized computer controlled Canon digital camera under ambient light as well as ultraviolet (302 nm) light emitted from a Wood's lamp (UVLMS-38 EL 3UV Lamp, UVP LLC; Upland, CA).

In vivo prevention of tumor establishment

Immediately following IP tumor inoculation (5x10^6 MSTO-211H-luc cells) and via separate injections, mice received IP treatments of either 140 mg/kg PTX as 34% PGC-PTX NPs (56 mg/kg PGC backbone), PGC-Bn NPs (58 mg/kg PGC backbone), 20 mg/kg PTX-C/E, 20 mg/kg PTX-C/E per day for 7 days, or 20 mg/kg PTX-C/E per week for 7 weeks (n=8/group). Tumor burden was evaluated via bioluminescence imaging (BLI) at 4 and 6 weeks after tumor inoculation (3 of the 8 mice per group being randomly selected at the beginning of the study). Images of mice were taken 10 minutes after subcutaneous injections of 2.25 mg firefly luciferin, using a Xenogen IVIS-50 bioluminescence camera (Caliper Life Sciences; Hopkinton, MA). The exposure setting was 60 s exposure, F4, small bin, and distance of 25 cm. All animals were monitored for tumor burden three times a week and were euthanized upon evidence of morbid disease including weight loss more than 15%, large palpable abdominal solid tumor, slow movement, and/or difficulty obtaining food and water. Overall survival was described by the Kaplan-Meier method and compared via log-rank test.

In vivo treatment of established tumor

We also studied PGC-PTX NP efficacy in an established tumor model[3]. Briefly, one week after IP tumor inoculation (5x10^6 MSTO-211H-luc cells), animals received one of the following IP treatments: 140 mg/kg PTX as 34% PGC-PTX NPs (56 mg/kg PGC backbone), PGC-Bn NPs (58 mg/kg PGC backbone), saline, or 20 mg/kg PTX-C/E per week for 7 weeks (n=8/group). Tumor

burden was evaluated via BLI at 4 and 6 weeks after tumor inoculation as previously described, and overall survival was described by the Kaplan-Meier method and compared via log-rank test.

Statistics

All data are reported as mean ± SD. Differences between groups were evaluated using one-way ANOVA, two-way ANOVA, or log-rank test using GraphPad Prism (GraphPad Software, Inc.; La Jolla, CA). Animals were randomly assigned to treatment groups. At the beginning of the *in vivo* efficacy studies, a subset of the animals (n=3) in each group was randomly chosen for subsequent BLI. No blinding was used in the animal studies.

References

1 H. Zhang and M. W. Grinstaff, *J. Am. Chem. Soc.*, 2013, **135**, 6806-6809.
2 M. Stolzoff, I. Ekladious, A. H. Colby, Y. L. Colson, T. M. Porter and M. W. Grinstaff, *Biomacromolecules*, 2015, **16**, 1958-1966.
3 R. Liu, A. H. Colby, D. Gilmore, M. Schulz, J. Zeng, R. F. Padera, O. Shirihai, M. W. Grinstaff and Y. L. Colson, *Biomaterials*, 2016, **102**, 175-186.

Supplementary tables and figures

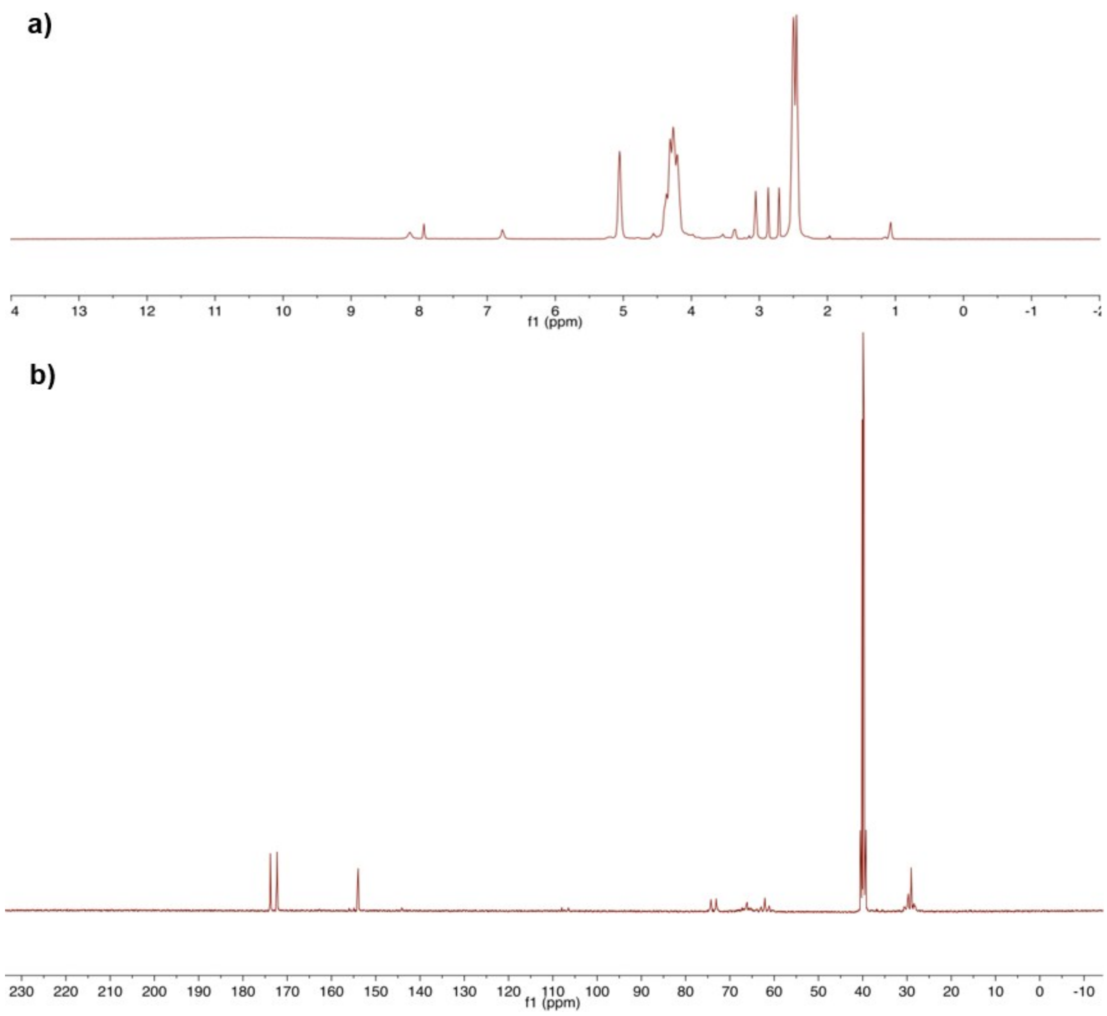

Fig. S1 Characterization of PGC-*g*-SA. (a) Proton and (b) carbon NMR spectra of PGC-*g*-SA. ^{1}H and ^{13}C spectra were acquired in DMSO-d_6 at 500 and 125 MHz, respectively.

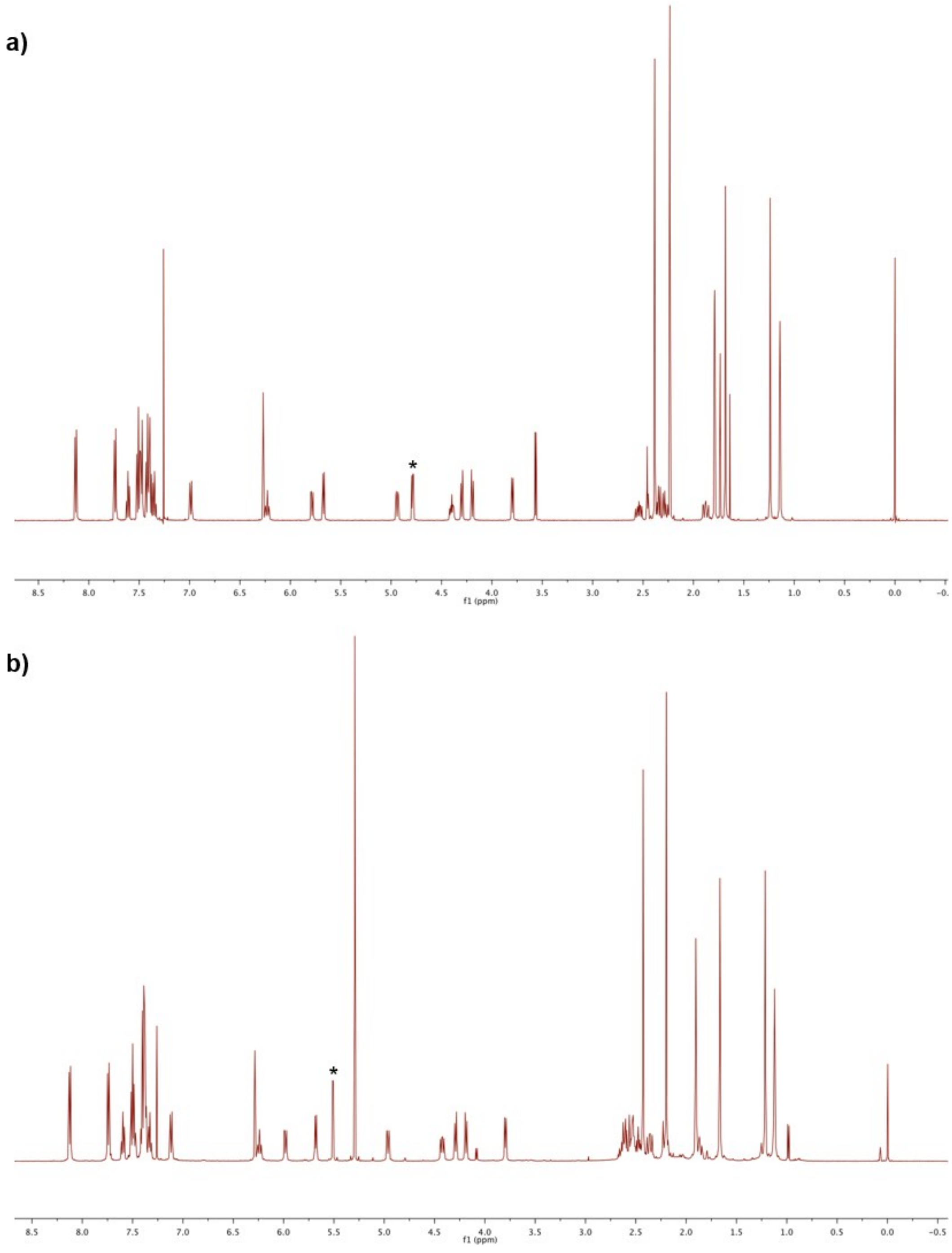

Fig. S2 Characterization of PTX-SA. Proton NMR spectra of (a) PTX and (b) PTX-SA acquired in chloroform-*d* at 500 MHz. After conjugation of SA, the PTX CH proton on C2’ (indicated by an asterisk) shifts from 4.79 to 5.51 ppm, confirming the conjugation of SA at the PTX C2’-OH.

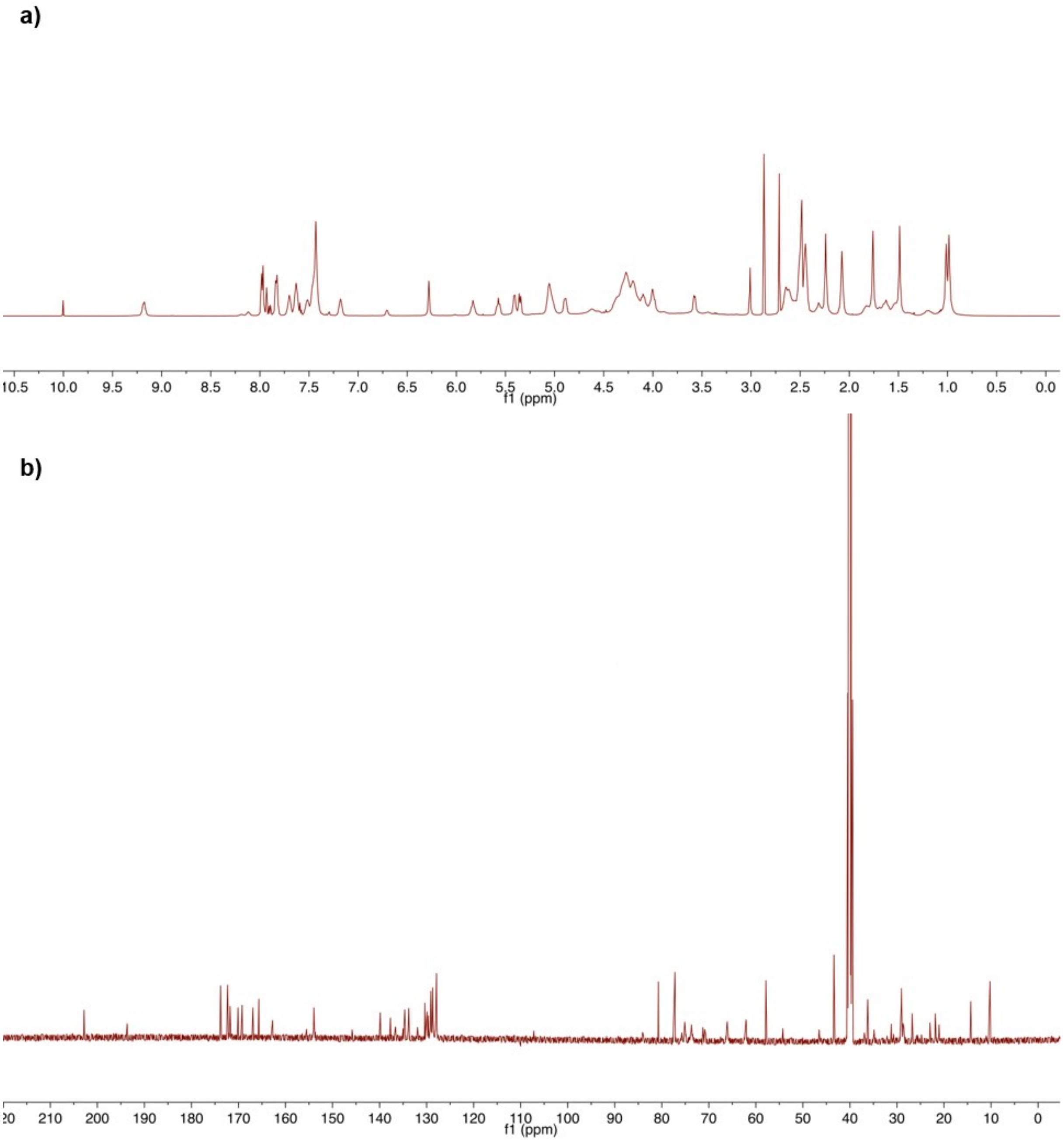

Fig. S3 Characterization of PGC-PTX. Sample (a) proton and (b) carbon NMR spectra of PGC-PTX acquired in DMSO-d_6 at 500 MHz.

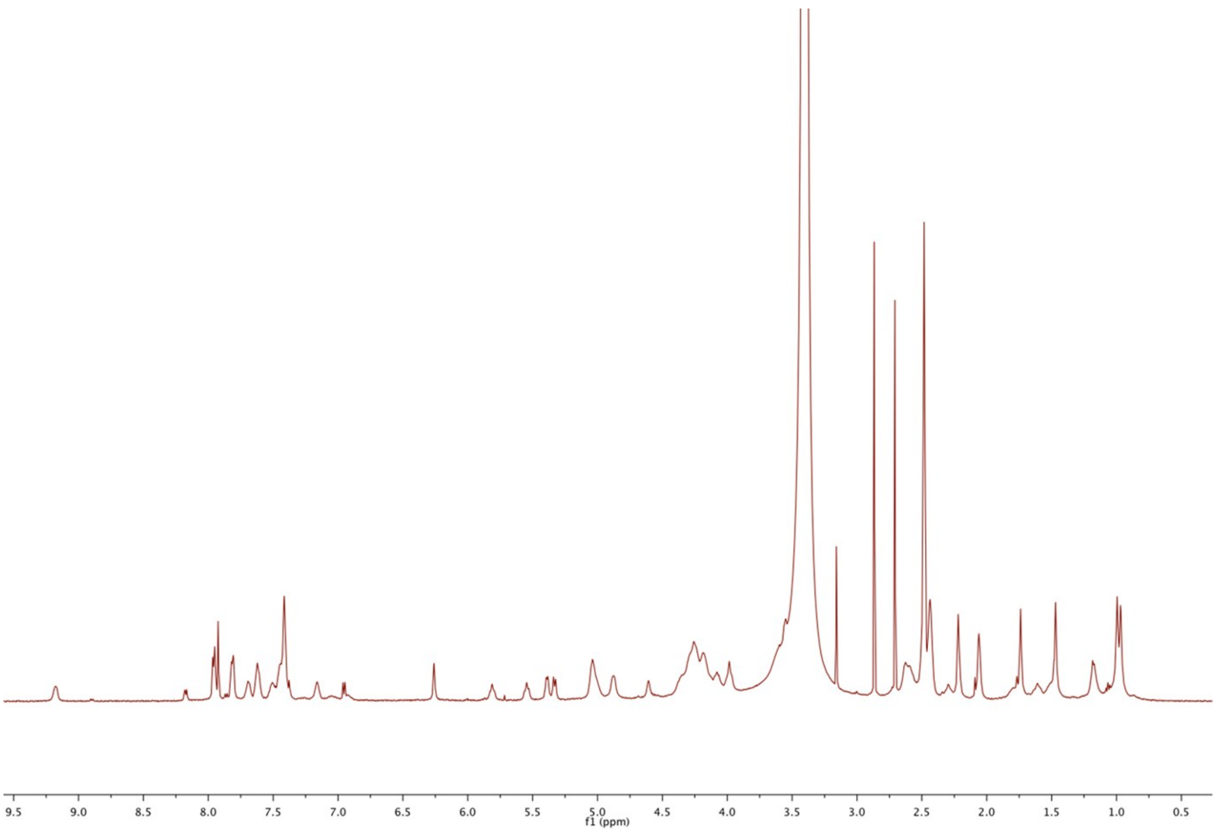

Fig. S4 Characterization of PGC-PTX-Rho. Proton NMR spectrum of PGC-PTX-Rho acquired in DMSO-d_6 at 500 MHz.

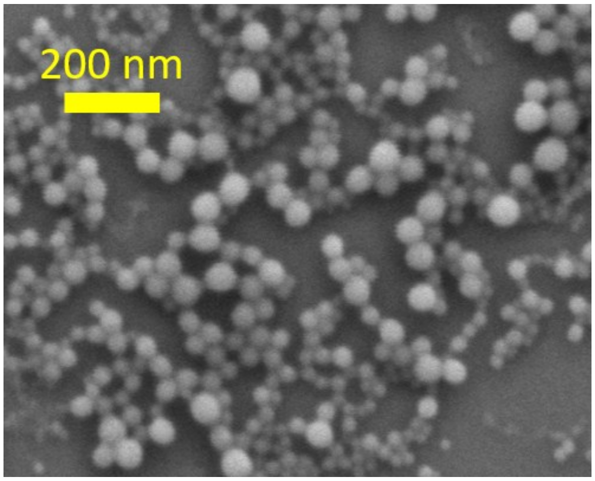

Fig. S5 70% PGC-PTX NP characterization. Scanning electron micrograph of PGC-PTX NPs with 70 mol% (74 wt%) PTX loading shows a polydisperse particle population with NPs between 10 – 90 nm.

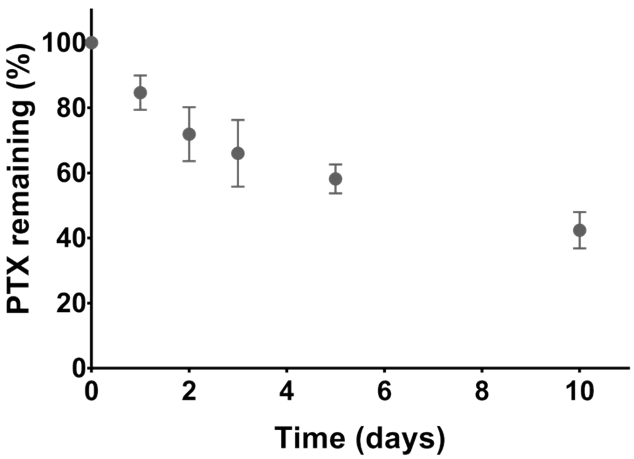

Fig. S6 Degradation kinetics of free PTX. The degradation of PTX was evaluated at 37°C in 10 mM pH 7.4 phosphate buffer supplemented with 0.3% SDS. Data is presented as the mean ± SD of three experiments.

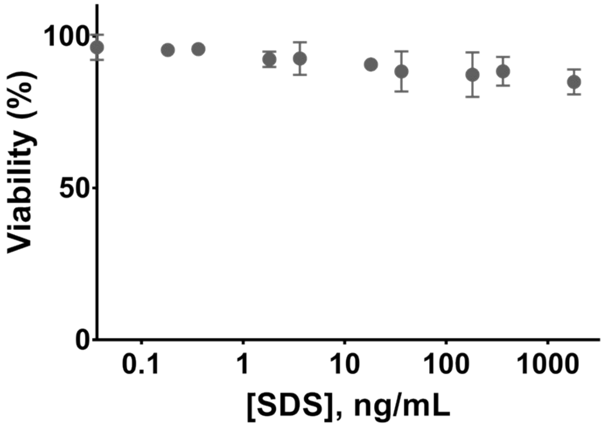

Fig. S7 SDS cytotoxicity in MSTO-211H cells. Cells were treated with SDS at equivalent concentrations to those employed in the 34% PGC-PTX NP formulation, and viability was assessed after 5 days of exposure. Data is presented as the mean ± SD.

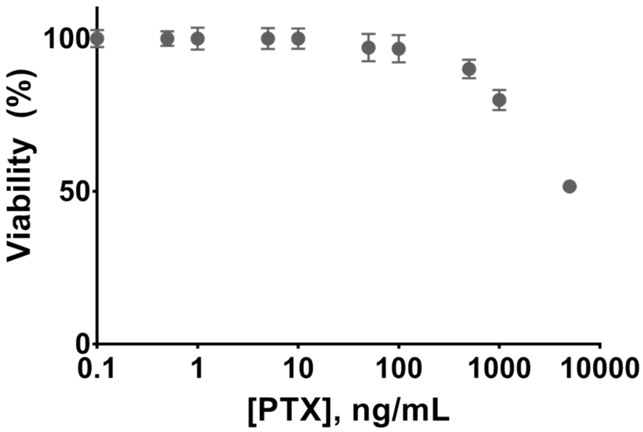

Fig. S8 MSTO-211H cell viability 24 h after treatment with 40% PGC-PTX NPs. At PTX doses ≤ 100 ng/mL, cell viability exceeds 95%. Data is presented as the mean ± SD of three experiments.

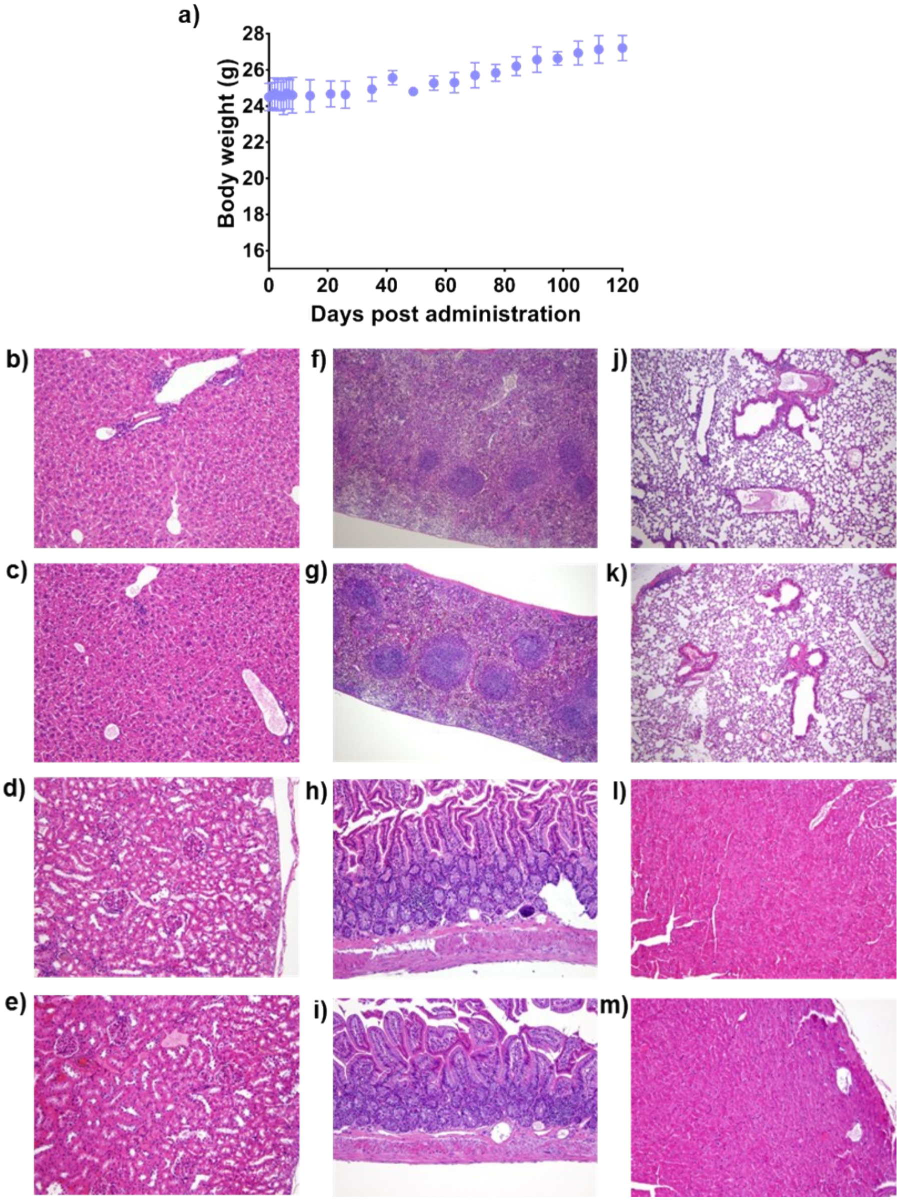

Fig. S9 Evaluation of 34% PGC-PTX NP safety. Healthy mice received 34% PGC-PTX NPs at a dose of 140 mg/kg PTX via IP administration. (a) Animals maintained healthy body weight during the 120 day survey period. (b,c) Liver (20x), (d,e) kidney (20x), (f,g) spleen (10x), (h,i) intestine (20x), (j,k) lung (10x), and (l,m) heart (20x) tissue were harvested from (c,e,g,i,k,m) treated animals and compared with (b,d,f,h,j,l) untreated controls via histological evaluation. The tissues harvested from the treated animals showed no pathologic changes, and were indistinguishable from the untreated healthy controls.

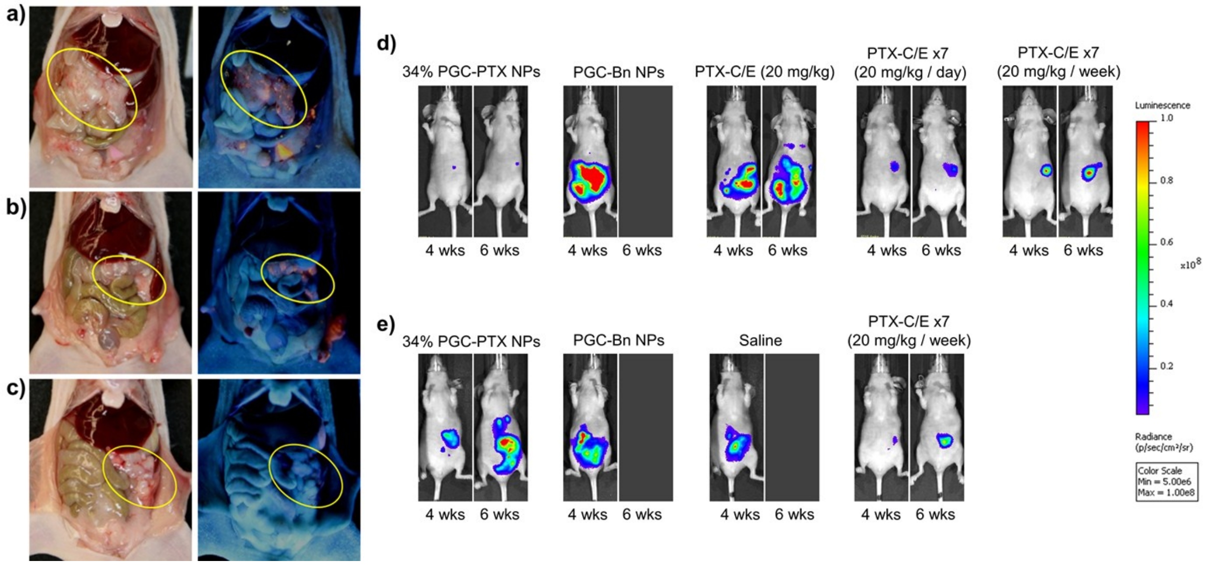

Fig. S10 Peritoneal distribution and efficacy of PGC-PTX NPs. To evaluate NP peritoneal distribution, mice received either (a,b) PGC-PTX-Rho NPs or (c) rhodamine in saline three weeks after IP tumor inoculation. The peritoneum was assessed under (left) ambient and (right) ultraviolet light 72 hours after injection. The largest tumors are circled in yellow. PGC-PTX-Rho NPs are visualized primarily in areas of IP tumor under ultraviolet light. PGC-PTX NP efficacy was evaluated in two models of peritoneal mesothelioma: (d) prevention of tumor establishment, and (e) treatment of established tumor. Tumor burden was evaluated for 3 randomly selected animals per group at 4 and 6 weeks after tumor inoculation via BLI. Sample BLI images are shown for one animal per treatment group.

Table S1 Size exclusion chromatography characterization of PGC-Bn and PGC-PTX with varying PTX loadings in mol%.

	Mn	PDI	Mp	Mw
**PGC-Bn**	13868	1.29	15618	17860
**34% PGC-PTX**	9393	1.35	11072	12651
**39% PGC-PTX**	10927	1.43	11770	15671
**43% PGC-PTX**	12324	1.56	13911	19237

Note: Molecular weights noted in Daltons.

Table S2 In vitro IC_{50} of PTX-C/E, PTX-SA-C/E, and PGC-PTX NPs with varying PTX loadings in mol% after 5 days of treatment.

	MSTO-211H	A549	PANC-1
**PTX-C/E**	9.42	1.76	2.98
**PTX-SA-C/E**	33.02	9.36	10.50
**34% PGC-PTX NPs**	107.40	21.75	23.46
**39% PGC-PTX NPs**	207.80	31.68	37.51
**43% PGC-PTX NPs**	463.80	61.80	41.48

Note: Concentrations noted in ng/mL PTX.
